# Impact of root canal preparation size and taper on coronal-apical micro-leakage using glucose penetration method

**DOI:** 10.4317/jced.51452

**Published:** 2014-10-01

**Authors:** Mehdi Tabrizizadeh, Maryam Kazemipoor, Seyed-Hossein Hekmati-Moghadam, Roqayeh Hakimian

**Affiliations:** 1 DDS, MSc, Associate Professor. Department of Endodontics, School of Dentistry, Shahid Sadoughi University of Medical Sciences, Yazd, Iran; 2 DDS, MSc, Assistant Professor. Department of Endodontics, School of Dentistry, Shahid Sadoughi University of Medical Sciences, Yazd, Iran; 3 DDS, MSc, Associate Professor. Department of Surgical and Clinical Pathology, School of Medicine, Shahid Sadoughi University of Medical Sciences, Yazd, Iran; 4 MA, Librarian and search literature officer. Department of Endodontics, School of Dentistry, Shahid Sadoughi University of Medical Sciences, Yazd, Iran

## Abstract

Objectives: The purpose of this in vitro study was to assess the effect of root canal preparation size and taper on the amounts of glucose penetration. Material and Methods: For conducting this experimental study, eighty mandibular premolars with single straight canals were divided randomly into 2 experimental groups of 30 samples each and 2 control groups. Using K-files and the balance force technique, canals in group 1 were prepared apically to size 25 and coronally to size 2 Peesoreamer. Group 2 were instrumented apically and coronally to size 40 and size 6 Peesoreamer, respectively. Rotary instrumentation was accomplished in group 1; using size 25 and .04 tapered and in group 2, size 35 and .06 tapered Flex Master files. Canals were then obturated by lateral compaction of cold gutta-percha. Glucose penetration through root canal fillings was measured at 1, 8, 15, 22 and 30 days. Data were recorded as mmol/L and statistically analyzed with Mann-Whitney U test (P value=. 05). Results: In comparison to group 1, group 2 showed significant glucose leakage during the experimental period (P value < .0001). Also, in each experimental group, the amount of micro-leakage was significantly increased at the end of the study. Conclusions: Under the condition of this study, the amounts of micro-leakage through root canal fillings are directly related to the size and taper of root canal preparation and reducing the preparation size may lead to less micro-leakage.

** Key words:**Dental leakage, root canal preparation, endodontics.

## Introduction

Chemo-mechanical or biomechanical preparation of the root canal system is recognized as being one of the most important phases in endodontic treatment (1,2). Shaping of the root canal system not only assist to obtain the biological objectives such as bacterial reduction, but also facilitate placement of a high quality root filling (3). According to Schilder (4) canals should be prepared as continuously tapering funnel shape from the apex to the access cavity and the apical opening should be kept as small as practical.

There is no consensus between who prefer large apical preparations for better removal of infected dentin and to promote the effectiveness of irrigants and who advocate smaller apical preparation combined with tapered shapes (5).

Enlargement of the canals by removing more dentin form the canal walls, may lead to preparation errors such as zips, canal transportations and perforations (6). Moreover, one of the potential factors, which may increase the risk for vertical root fracture, is the prepared canal diameter (7). Although, greater tapers may decline the root stresses occurred at the time of root canal filling, but these stresses increase during masticatory loadings (8).

The influence of final apical preparation size on treatment outcome has been evaluated in two long – term studies. Kerekes and Tronstad (9) reported similar results for prognosis in apical preparation to ISO sizes 20-40 and 45-100. Whereas Strindberg (10) found a poorer prognosis for larger apical preparation. Also, the results of a Toronto study (11) on endodontic outcomes favored smaller preparations in comparison to larger apical shapes (90% and 80% success, respectively). A randomized controlled trial (12) evaluated the effect of apical preparation size on the outcome of primary endodontic treatment. According to this study, the enlargement of the canal to 3 sizes larger than FABF (First Apical Binding File) is adequate, and further enlargement does not provide any additional benefit during root canal therapy.

Periradicular inflammation is the consequence of a coronal and ⁄ or apical pattern of fluid and microorganism flow (micro-leakage) (13). Although, many attempts have been made to resolve the sealing problems in root canal system, they have failed to eliminate this phenomenon completely (14).

Various test methods such as dye penetration (15), radioactive isotopes method (16), bacteria or bacterial metabolites leakage test (17); electrochemical technique (18), gas chromatography (19), and fluid filtration (20) have been introduced for detection of micro-leakage.

Recently a new “glucose penetration” model was described to evaluate endodontic micro-leakage (21). Xu, *et al.* (21) introduced this method for analysis of endodontic micro- leakage. This method is based on the filtration rate of glucose along the root canal filings (21). Glucose has a small molecular size (MW=180Da), is a nutrient for bacteria, hydrophilic and chemically stable. Therefore, this method is more clinically relevant than other micro-leakage tests (21).

In addition to the aforementioned contributing factors in micro- leakage such as sealer type, obturation technique and etc.; it seems that there is another important factor: size of the canal preparation. Surprisingly, evaluation of the effect of canal preparation size, both apically and coronally, on the micro- leakage is overlooked.

The purpose of this in vitro study was to compare micro-leakage through root canal filling material using different sizes and tapers. Moreover; the majority of the studies on micro-leakage are accomplished with dye penetration test that has many limitations. In this study, micro-leakage was assessed by a recently introduced method: glucose penetration. Understanding of the construction and performance of the glucose penetration set up may be useful in the future studies to assess micro-leakage.

## Material and Methods

- Sample Selection

Eighty freshly extracted human mandibular premolar teeth, with a single straight root canal, were selected for this study. All teeth had intact coronal surfaces with closed apices. Any calculus and soft tissues were removed from the root surfaces, and then teeth were decoronated at 14mm from the apex. Prior to root canal preparation, all samples were autoclaved (Euroklav 23 V-S, Melag, Germany) for 40 min at 121°C and 15 psi in autoclave bags.

- Root Canal Preparation

After removal of the pulp tissue using a barbed broach, the working length was determined by inserting a size 15k-file (Dentsply, Maillefer, Ballaigues, Switzerland) into the root canal until the tip was just visible at the major apical foramen. The root canals were prepared 1mm short of this length. Only the teeth with the adapted initial size 15 file were included in the experiment.

The patency of the apical foramen was ascertained by inserting the tip of a size 15 file through it, before and after the root canal preparation. The teeth were then randomly divided into 2 experimental groups of 30 roots each and 2 control groups of 10 each.

Group1 (n=30): The apical portion of the canal was prepared with the balanced force technique (21) and k-file to a size 25 master file. After every instrumentation, canals were rinsed with 1ml freshly prepared 2.5% sodium hypochlorite (NaOCl) solution and a 30-gauge needle (Supa, Tehran, Iran).

The Coronal portion of the canal was then flared with size2 Peeso reamer (Dentsply, Maillefer, Ballaigues, Switzerland) that created a space with 0.9 mm diameter at this area. The Peeso reamer drill was penetrated to the canal to the depth of the cutting flutes. Flaring was followed by irrigation with 1ml of 2.5% NaOCl.

Rotary NiTi Flex-Master files (VDW, Munich, Germany) were used in an electric handpiece (Endomate 2, NSK, Japan) set to 400 rpm for completion of the instrumentation. Rotary instrumentation was performed using a size size25 with .04 tapered instruments (with 0.77mm diameter at 14mm from the file tip) and a crown – down technique.

A typical sequence was as follows: .04-20, 02-20 until the advancement to the working length with a size 20 instrument. The canals were then enlarged to size 25 using instruments with the same taper. Preparation continued with the size size 25 file of sequentially larger tapers until the final apical file size was achieved. After every third file, canals were irrigated with 1ml of 2.5% NaOCl.

After biomechanical preparation, a final rinse with 10 ml 17% EDTA (pH=7.7) (Surdent, Germany) and 10ml 2.5% NaOCl was given for 1 minute, to remove the smear layer. Subsequently, canals were irrigated with 5ml deionized water to eliminate any residual chemical effects of irrigating solutions.

Group2 (n=30): The apical portion of the canal in this group was prepared to a size 40 master file. Peesoreamer drills size2-6 were then used to flare the coronal part of the roots in the manner that each successively larger drill penetrates 1 to 2 mm short of the previous size into the canal. Peesoreamer size6 was only inserted to the depth of the cutting flutes. The final objective file in the rotary instrumentation was .06 taper size size35.

- Obturation

Canals in the first and second groups were dried with size 25 and 40 paper points respectively. Afterward, canals were filled with gutta- percha (Diadent, Korea) and AH26 (Dentsply, Maillefer), using cold lateral compaction method. In group 1, a size 25 master gutta – percha (GP) cone and in group 2 a size 40 master GP cone lightly coated with sealer and then inserted into the canal to the full working length. Appling a size B finger spreader (Dentsply, Maillefer) and size 15 accessory cones, lateral compaction was performed to a level approximately 1mm short of the working length. Each accessory cone (Diadent, Korea) was coated with sealer and then placed into the canal until the spreader could not penetrate more than 2 to 3mm. The excess gutta –percha, at the root canal orifice, was removed with a heat carrier (Dentsply, Maillefer, Switzerland) and the filling was compacted vertically.

- Positive and Negative Control Groups

The positive control group (n=10) consisted of canals that were prepared as the experimental groups (5 teeth for each group); however obturation was rendered without any sealer. The negative control teeth (n=10) were prepared as the same as the positive controls and obturated with GP cones and AH26 as sealer.

All teeth were stored in an aqueous solution containing 0.1% sodium azide (NaN3) for 1week to avoid samples recontamination and to allow the sealer to set. Roots in the experimental groups and the positive controls were coated with nail varnish, except for the root canal orifices and apical apices. Root surfaces in the negative control group were completely covered with nail varnish.

- Model Assembly: Glucose Penetration Model

We applied a modified glucose penetration set up as describes below:

The end of a micro-tube (Eppendorf co., Hamburg, Germany) was cut and coronal part of each root was glued through it using sticky wax. Leakage at this connection was eliminated by the generous use of cyanoacrylate glue. The caps of the micro-tubes were removed using a diamond disk. A plastic tube, 14cm long, was connected to the coronal portion of each micro-tube and then, seal was obtained using cyanoacrylate glue and sticky wax.The assembly was then placed in a sterile 5 ml test tube (Eppendorf co., Hamburg, Germany). Drilling a uniform hole in the upper part of test tubes created an open system.

The upper chamber contained 14cm of 1mol/L glucose and 0.2% NaN3 solution (approximately 20 ml), which created a hydrostatic pressure of 1.5kpa (15cm H2O) 21. Glucose that leaked through the obturating materials, were collected in the lower chamber that contained 4 ml 0.2% NaN3 solution. Connecting the open end of the plastic tube to compressed air checked the seal of the system. Seal of the apparatus may be questioned, if any bubble was observed.

Throughout the experimental period, the whole setup was stored at the room temperature. Liquid level, in both upper and lower chambers, was marked and controlled every 2 days. Any evaporation was compensated by addition of sterile deionized water to the solutions to reach the initial volume

- Spectrophotometer Measurements

A total volume of 10 µL was taken from the solution in the test tube, using a digital micro-pipette (Biohit, Lab-tron, Iran) at 1, 8, 15, 22 and 30 days. Afterward, 10 µL of freshly 0.2% NaN3 was added to the test tubes to keep constant volume of 4 ml in the lower chamber. The sample was then analyzed with a Glucose assay kit (Pars Azmun co., Tehran, Iran) and a spectrometer (Clinic II – PHOTOMETER, Tajhizat Sanjesh, Iran) at 546 nm wavelengths.

The detection limit of glucose assay was 5 - 400 mg/dL. The values below 5 mg/dL recorded as 0(no leakage) and concentrations over 400 mg/dL were diluted to reach the exact concentration values.

- Statistical Analysis

Since the data were not normally distributed (Shapiro-Wilk test), determining the significant difference between groups was rendered through pair wise comparisons and Mann-Whitney U test. The level of statistical significance was assigned at *P* value =. 05.

## Results

**Figure 1:**
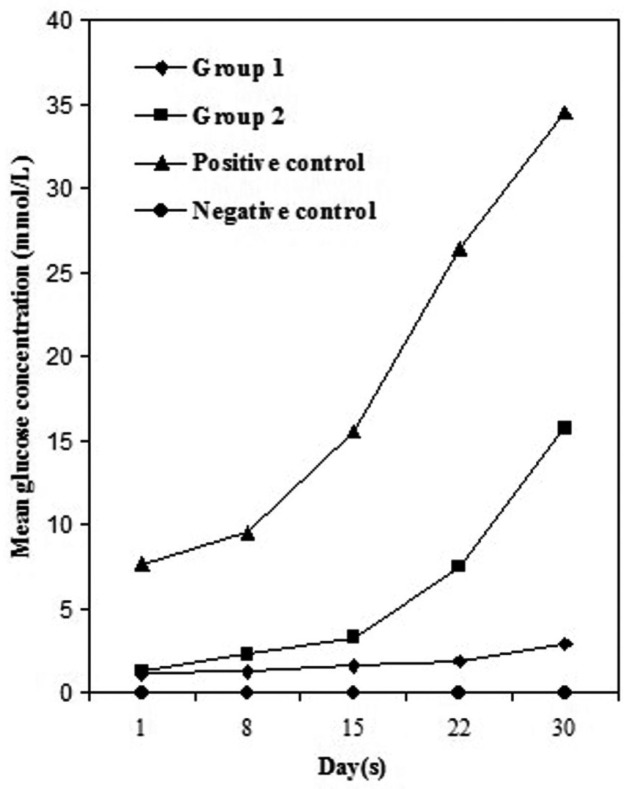
Mean glucose concentration in mmol/L throughout the experimental period (days).

The results of this study are summarized in  Table 1 and figure 1. The negative control group showed no glucose penetration during the experimental period. This implies on the reliability and effectiveness of seal in our leakage evaluation set up. As contrast, the positive controls showed significantly more leakage in comparison to experimental groups at every time interval defined in this study (*P* value<.001).

**Table 1:**
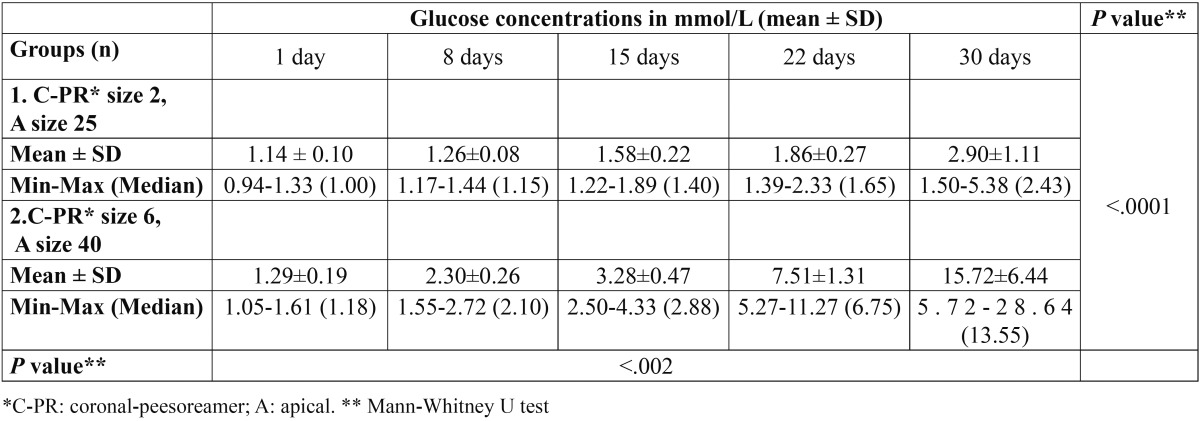
Glucose concentration in mmol/L during the experimental period (mean ± SD)

Although the amounts of glucose penetration of two experimental groups increased over time but the manner of this increase was different in two groups. Group 2 showed significant increase in leakage values over time and a rapid increase in glucose penetration was observed after day 15. The amounts of leakage in Group 1 were increased in a slower manner throughout the study. Group 2 showed highly significant difference in leakage values in comparison to group 1 (*P* value<.0001).

The Mann-Whitney U test demonstrated that there was a significant difference between the two experimental groups at the 1st, 8th, 15th, 22nd, and 30th days (*P* value<.002).

## Discussion

Although microbiological studies have shown that larger apical preparation sizes may lead to a greater reduction in remaining bacteria (2), but there are two concerns in this regard: the impact of final canal shape and size on root strength and the impact of apical enlargement on the outcome of endodontic treatment.

Removing more dentine from the canal walls during instrumentation, creates an irregular dentin walls, significantly weaken the root and may predispose canal for vertical root fracture (8,22). Considering the apical enlar-gement, most authors have found that there was no difference in healing rate after root canal therapy (9).

Microleakage phenomenon both apically and coronally may lead to periradicular inflammation. Various factors (e.g. the presence or absence of smear layer, root canal obturation techniques, physical and chemical properties of sealers) may affect the apical leakage but the impact of canal preparation size on micro-leakage has not been assessed up to the present.

In the present study, we evaluated the effect of canal size, both coronally and apically on the amount of micro-leakage using a new quantitative method: glucose penetration. Since leakage occurs through two surface areas, increasing the surface area may lead to an increase in micro-leakage.

The results of our study demonstrated that the two sizes of preparation showed significantly different levels of leakage throughout the experimental period. According to our results, all canals showed low degrees of leakage at day 1. During the experiment; we observed a little increase in leakage values in group 1(small preparation size) . The amounts of leakage in group 2 increased slightly at day 8 and day 15. At day 22, there was a rapid increase in leakage values in group 2 and this tendency to increase continued to the end of the experimental period.

Micro-leakage studies, in vitro, are mostly based on the detection of a tracer that would penetrate through the obturated canal (13). “Glucose penetration” model is a newly introduced method for evaluation of the micro-leakage (21). The superiority of this technique, over other leakage detection tests, is attributed to the tracer that used possibility to analyze leakage quantitatively and continuously over time and its sensitivity and reproducibility (21). Various factors may affect the results obtained from glucose penetration method.

As glucose is a nutrient for bacteria, the presence of bacteria in the canal or recontamination of the specimens during the study may impact on glucose concentration. It has been recommended that, avoiding cross contamination in micro-leakage and bonding studies in vitro, it is better to autoclave samples rather than storage in formalin (23). Heat and pressure did not alter the molecular structure of collagen and dentin permeability, but may impact on the formation of hybrid layer that leads to a slight increase in leakage values (23). In comparison to other sterilization methods, autoclaving did not significantly effect on the result of micro-leakage tests (23). Higher values of glucose concentration that were observed in our study(in comparison to Xu, *et al.* (21) findings) may be partly due to the increasing effect of sterilization process on the amounts of leakage and lack of existing bacteria in root canal environment for glucose consumption.

Glucose molecule is very unstable and would breakdown rapidly. Sodium azide solution was used as the storage medium in this study to preserve this molecule during the experiment. This solution also has an antibacterial effect and could prevent recontamination of samples throughout the experiment.

The quality of seal after root canal obturation is directly related to the prepared canal shape (24). In this study, canals were prepared with a combination of hand and rotary instruments. Compared to stainless steel hand files, NiTi rotary instruments are more effective in their shaping ability, time consuming and create a suitable taper with smooth dentinal walls (24).

Except for” Balance force” technique, K – files performed inferior in comparison to NiTi rotary in vitro (24). During instrumentation, reaming motion creates a rounder canal shape that is suitable for adaptation of GP cones to the canal walls and promotion of seal (24).

Smear layer removal may also impact on the amounts of coronal micro-leakage in vitro (25). Independent to the obturation technique, removing the smear layer from canal walls, leaves more patent dentinal tubules, permits penetration of sealer into open dentinal tubules, improves the quality of seal and reduces micro-leakage (25). Considering that combined application of NaOCl and EDTA for irrigation decreases the coronal micro-leakage (25), we have used this regimen in our study.

The potential of glucose reactivity with the obturation materials could interfere with the micro-leakage values. It has been shown that Resilon, Epiphany, GP and AH26 did not affect the glucose concentration over time (26). Conversely, Portland cement, MTA, Ca(OH)2 and Sealer 26 could react with glucose solution (26).

Technical aspects of glucose penetration set up such as the hydrostatic pressure of glucose solution can influence the results. According to Pommel and Camps (27) for measuring the leakage through root canal fillings, low (15cm H2O) and high (150cm H2O) pressures did not differ significantly. The hydrostatic pressure of a liquid is dependent to the density and height of liquid column. Similar to other studies that rendered with glucose penetration method, we have applied low coronal pressure in our study.

The presence of entrapped air or void may also affect the glucose penetration method. Özok, *et al.* (28) demonstrated that their glucose penetration set up could detect “a true void (a through-and-through void that communicates with both ends of the filling) but a cul-de-sac type void (porosity) might remain undetected.” Method of root canal obturation and ratio of GP to sealer would determine the frequency of voids (28). Conventional cold lateral compaction and warm vertical compaction in combination with either AH26 or AH Plus sealer may decline the frequency of voids significantly (29). Another concern in micro-leakage studies is comparability and reproducibility of the results that obtained from different micro-leakage tests. Previous studies mostly showed poor or no correlation between different microleakage tests other than glucose penetration method (13-15). Sauza, *et al.* (30) in their study concluded that leakage results in the fluid transport model and the glucose penetration model were similar.

Previously performed leakage studies with glucose penetration method (21,30) did not address the influence of canal preparation size on the amounts of micro-leakage. Different leakage values that were observed in this study may be the consequence of specimens’ sterilization, size of canal preparation, difference in surface areas that leakage occurred through and type of the applied sealer. As mentioned above, there are many factors that could interfere with the results of glucose penetration model. Thus, to minimize the effect of these intervening factors, it is necessary to uniform the leakage studies with glucose penetration method in this regard.
